# Protective Geopolymer Coatings Containing Multi-Componential Precursors: Preparation and Basic Properties Characterization

**DOI:** 10.3390/ma13163448

**Published:** 2020-08-05

**Authors:** Chenhui Jiang, Aiying Wang, Xufan Bao, Zefeng Chen, Tongyuan Ni, Zhangfu Wang

**Affiliations:** 1 Key Laboratory of Marine Materials and Related Technologies, Zhejiang Key Laboratory of Marine Materials and Protective Technologies, Ningbo Institute of Materials Technology and Engineering, Chinese Academy of Sciences, Ningbo 315201, China; 2 Hongrun Construction Group Co., Ltd., Shanghai 200235, China; xfbao_hr@126.com; 3 Department of Construction Engineering, Zhejiang College of Construction, Hangzhou 311231, China; 4 Zhejiang Huawei Building Materials Group, Co., Ltd., Hangzhou 310018, China; chenzf_11747@126.com (Z.C.); piter_wang1968@126.com (Z.W.); 5 College of Civil Engineering, Zhejiang University of Technology, Hangzhou 310023, China; hznity@zjut.edu.cn

**Keywords:** geopolymer coatings (GPC), setting time, compressive strength, adhesive strength, impermeability

## Abstract

This paper presents an experimental investigation on geopolymer coatings (GPC) in terms of surface protection of civil structures. The GPC mixtures were prepared with a quadruple precursor simultaneously containing fly ash (FA), ground granulated blast-furnace slag (GBFS), metakaolin (MK), and Portland cement (OPC). Setting time, compressive along with adhesive strength and permeability, were tested and interpreted from a perspective of potential applications. The preferred GPC with favorable setting time (not shorter than 120 min) and desirable compressive strength (not lower than 35 MPa) was selected from 85 mixture formulations. The results indicate that balancing strength and setting behavior is viable with the aid of the multi-componential precursor and the mixture design based on total molar ratios of key oxides or chemical elements. Adhesive strength of the optimized GPC mixtures was ranged from 1.5 to 3.4 MPa. The induced charge passed based on a rapid test of coated concrete specimens with the preferred GPC was 30% lower than that of the uncoated ones. Setting time of GPC was positively correlated with *η*[Si/(Na+Al)]. An abrupt increase of setting time occurred when the molar ratio was greater than 1.1. Compressive strength of GPC was positively affected by mass contents of ground granulated blast furnace slag, metakaolin and ordinary Portland cement, and was negatively affected by mass content of fly ash, respectively. Sustained seawater immersion impaired the strength of GPC to a negligible extent. Overall, GPC potentially serves a double purpose of satisfying the usage requirements and achieving a cleaner future.

## 1. Introduction

Geopolymers, also known as inorganic polymers or alkali-activated materials (AAMs), are a cluster of materials synthesized with alkali-activation and subsequent geopolymerization at elevated or ambient temperature [1,2,3]. Davidovits distinguished geopolymers from AAMs in that the both pertain to different systems, respectively, from the perspective of material science [4]. Due to industrial by-products and/or other inexpensive aluminosilicate sources such as fly ash (FA), granulated blast furnace slag (GBFS), steel slag, metakaolin (MK), waste glass, saw dust, mine tailing, rice husk ash, natural pozzolans, and water glass (WG) can be potentially utilized in production of geopolymers [5]; a large quantity of non-renewable resources stands a chance of conservation as well as massive solid waste is recycled and reused. Less greenhouse gas emission can be achieved by replacing traditional cement-based materials with geopolymer-based alternatives in building industry and civil engineering sector [6]. This means that geopolymer-based materials often lead to less energy consumption and environmental footprint.

Furthermore, geopolymer-based materials possess comparable or even superior mechanical properties and durability, especially excellent resistance to chemical corrosion and thermal properties [7,8,9,10,11,12,13,14]. Research findings revealed that geopolymers blended with various mineral additives show a significant improvement in mechanical properties and durability at various temperature conditions [7,8]. The results advocate that geopolymer mixtures with desired properties can be designed for ambient temperature curing condition with minerals additives, which may further promote them as an environmentally friendly construction material [7,8,9]. Geopolymer-based binders show inherently superior fire resistance as compared to Portland cement-based binders. However, it requires careful choice of precursor, use of aggregates, total alkali content in geopolymer, water content, and so forth [10]. The production of geopolymer concrete requires great care and correct material composition [10,11]. During the activation process in making the geopolymer, high alkalinity also requires safety risk and enhanced energy consumption and generation of greenhouse gases [11,14]. Geopolymer concretes using aggregates of different reactivity are reported to expand less than the corresponding Portland cement-based concretes [12,13]. The ability to utilize alkali-silica reaction vulnerable aggregate in the production of geopolymer concrete would increase economic and sustainability appeal [13,14].

Hence, as typical of cleaner and sustainable materials, geopolymers are expected to be powerful and cleaner products with promising prospects in the near future. They have been attracting more and more attention worldwide. Today the primary application of geopolymers in civil engineering is the development and production of building materials as a potential alternative to traditional materials with high energy consumption and pollution [15,16,17,18]. Geopolymers are expected to be green binding material and consume less energy. Production of geopolymer minimizes waste production and protect environment [15,16]. Geopolymers/alkali-activated binders have attracted considerable attention as promising construction and repair materials since their discovery because of their superior properties. Moreover, less pollution was caused by geopolymer/alkali-activated concretes than conventional cement concretes [17,18]. However, further research is needed to identify the sustainability and low-carbon nature of geopolymers and related products [1,3,5].

For the purpose of prolonging the lifespan of civil structures, sealing the exposed surface of concrete with coatings has been widely studied and utilized. Surface protection/treatment has become more significant in marine concrete structure, which is vulnerable to chemical deterioration and physical damage. Although organic coating materials (e.g., epoxy resins, silane and acrylic) have been commonly used, there exist disadvantages and drawbacks involving ease to crack/peel/degradation, inability to release vapor pressure [19], susceptible to fire [20], release of odors, lack of stability under UV radiation [21], difficulty to be removed after aging [22] (Pan et al., 2017), and low resistance against thermal shock [23]. In comparison, as previously reported by Franzoni [24] and Jiang [25], inorganic coatings possess desirable adhesive strength, good durability and favorable compatibility with concrete substrate. In addition, geopolymers show inherently superior fire and thermal shock resistance [26]. However, less attention and interest has been concentrated on this field, especially with regard to their penetration depth, bonding interface, interactions with substrate [22], and transport mechanism of deleterious ions in this type of coating layer.

As an innovative inorganic material with excellent performance, geopolymer coatings (GPC) can also be potentially used for the protective layer of concrete structures. Zhang et al. presented an experimental study of employing geopolymer-based material as a coating material for marine concrete protection [27]. Aguirre-Guerrero et al. prepared alkaline-activated FA/MK geopolymer mortar as an interfacial agent that prevented reinforcing bars embedded in reduced-scale concrete members from corrosion [28]. Wiyono reported that surface soundness of pozzolan concrete can be enhanced through the application of GPC layer [29]. Lv and co-authors synthesized a powder GPC by mixing MK and solid water glass along with additives and fillers [30]. They claimed that the dry-mixed coating material has been successfully applied in indoor engineering. Wang and Zhao issued a tentative study on silica fume-based GPC for fireproof plywood that was modified with decanoic-palmitic eutectic mixture [31]. In addition, from a point of view of sustainability, the carbon footprint of GPC is several times lower than that of traditional organic-based coatings [32]. The GPC prepared from multi-componential precursors at ambient temperature and its applications on concrete substrate, however, has seldom been studied, thus hindering a better understanding towards potential utilization of geopolymers.

The objective of this study, hence, is to design and prepare green GPC mixtures comprised with a quadruple precursor at ambient condition and to correlate basic engineering properties of GPC to its several key influencing factors. This paper is structured as follows: In the first step, a series of GPC were formulated and prepared based on the proposed methodology. Secondly, setting time and mechanical strength alongside rapid chloride permeability of the prepared and optimized GPC mixtures were experimentally investigated. The correlations between these properties and key mixture parameters of GPC were interpreted. Moreover, mechanical strength of GPC cured in standard condition and in artificial seawater was compared. In the end, the adhesive strength of GPC to a typical concrete substrate was evaluated with a portable apparatus. As a complement, the potential sustainability of this type of geopolymer-based applications was briefly reviewed.

## 2. Experimental Program

### 2.1. Materials

The constituent materials used can be classified into three parts in terms of their functions, i.e., aluminosilicate precursors (or binders), alkali activators and additives. The precursor includes FA, GBFS, MK and ordinary Portland cement (OPC) as per Chinese standards and technical specifications. All raw materials used in the study are supplied by Zhejiang Huawei Building Materials Group, Co., Ltd., in Hangzhou, China. Compared to one single precursor, the quadruple combinations were expected to complement each other’s advantages with regard to chemical compositions, specific properties (e.g., particle size distribution and reactivity), economical cost, as well as sustainability. X-ray fluorescent (XRF) analysis (Olympus, Tokyo, Japan) was carried out to obtain oxide compositions of the precursors as shown in Table 1.

**Figure 1:**
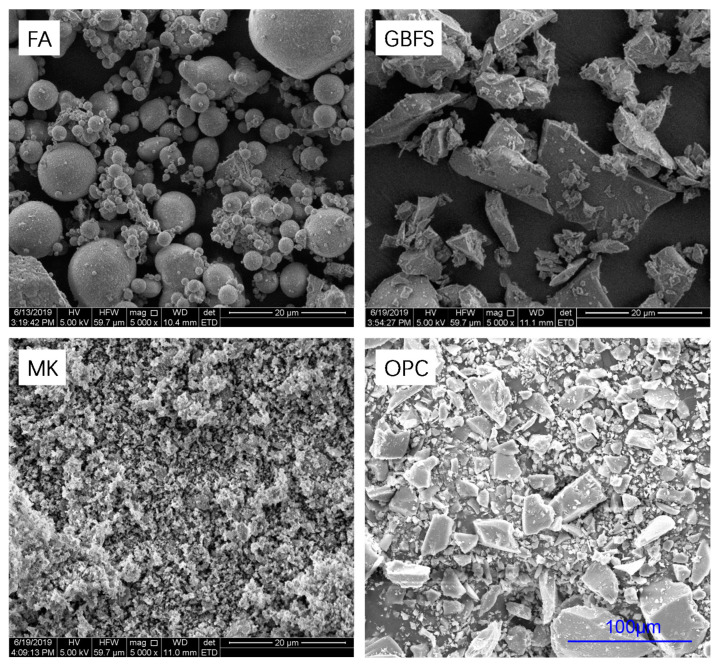
Scanning electron micrographs (SEM) of the precursors used.

**Figure 2:**
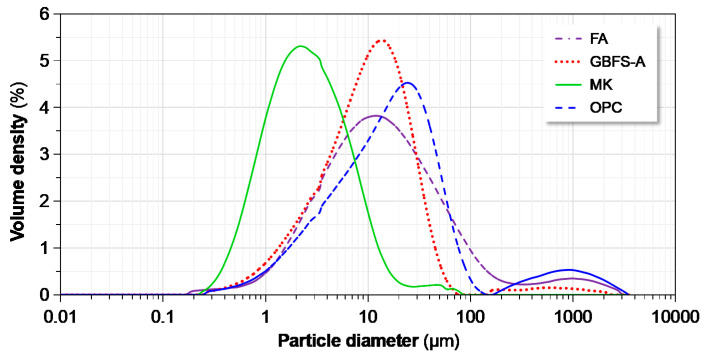
Particle size distributions (PSD) of the precursors employed in this study.

In order to make the GPC protective layer as compact as possible, the overall particle size distribution (PSD, Lab Synergy, New York, NY, USA) of the multi-componential precursors was optimized with the aid of EMMA (Version 1, Elkem, Olso, Norway) [33], a software package of particle packing calculation. Figure 1 and Figure 2 respectively show scanning electron micrographs (SEM, Nanoscience, Phoenix, AZ, USA) and the laser-based PSD of the precursors. The FA mainly consists of spherical particles of glass, while the GBFS, MK and OPC are all composed of irregular and angular particles that are similar with previously published results [34,35,36]. The MK possesses the finest particles in all the precursors used, as clearly shown in Figure 1 and Figure 2. It was expected to effectively fill spaces between coarser particles and densify the overall quadruple binder system. The GBFS and OPC with higher reactivity are beneficial to geopolymerization and development of mechanical properties of GPC at ambient temperature.

It is well-known that the geopolymerization process of geopolymer-based materials at ambient temperature is relatively slow and, therefore, a desirable strength is often difficult to obtain at early ages. Elevated temperature can accelerate the formation of geopolymer gel, and thus, enhance the strength. However, heat curing is often difficult to achieve in on-site applications. From the engineering and economical perspectives, therefore, it is essential to develop the GPC mixture with favorable mechanical strength under ambient condition. Based on the hypothesis that additional calcium silicate hydrate (C-S-H) gel coexists with geopolymer products, calcium-rich materials such as GBFS and OPC were introduced to shorten setting time and to improve mechanical properties of GPC. Additionally, heat release from hydration of the calcium-rich materials can accelerate the geopolymerization.

The alkaline activator was a blended aqueous solution of commercially available sodium hydroxide (NaOH pellets with 99.8% purity) and water glass (WG, with 27.5% SiO_2_, 8.7% Na_2_O and 63.8% H_2_O by mass) synthesized from industrial by-products. A polycarboxylate-based superplasticizer (SP, with 26.2% solid mass content and 25.8% water-reducing ratio) and polypropylene fiber (PPF, with length of 6–10 mm and aspect ratio of 400–600) were used as functional additives of GPC. The former is favorable to improve workability of fresh GPC mixture while the latter is beneficial to toughness and cracking resistance of the hardened GPC layer. It is noteworthy that, once cracking occurs, a penetration pathway for corrosive agents will be emerged in the GPC layer and the protected substrate will be deteriorated.

### 2.2. Experimental Parameters

In this experimental investigation, primary engineering properties involving setting time, compressive and adhesive strength, along with permeability of a number of GPC mixtures were explored and interpreted. It is essential that GPC set neither too rapidly nor too slowly. In the first case, there might be insufficient time to operate the fresh mixture before it becomes too rigid. In the second case, too long a setting period tends to delay the work unduly, also it might postpone the actual use of the coating layer because of inadequate strength at the prescribed age. Consequently, initial and final setting time of GPC must be designated in a definite and reasonable range.

Compressive strength is the most frequently-used parameter for characterizing the engineering properties of hardened geopolymer-based materials [37,38]. Adhesive strength refers to the capability of a GPC layer to stick to a substrate. It is often measured by assessing the ultimate tensile stress to detach or unstick the hardened GPC layer perpendicular to the substrate. It is essential that GPC possesses robust impermeability to moisture and deleterious substances. As a typical scenario, the penetration and transport of chloride ions often resulted in deterioration of concrete structures in marine atmosphere. As a trial and error, we employed electric charge passed based on rapid chloride permeability test to characterize the protection effect of GPC.

It is noteworthy to state that all test data in this article are average values from two or three replicate samples on the basis of identical experimental configurations, respectively. Relative standard deviations (i.e., the ratio of standard deviation to average value) of all the presented data in figures and tables are in the range of 3–8%. The scatter of test data is totally lower and meets the requirements of experimental design. For these reasons, the error bars and statistical analyses of the experimental results were uniformly omitted.

### 2.3. Mixture Formulation

**Figure 3:**
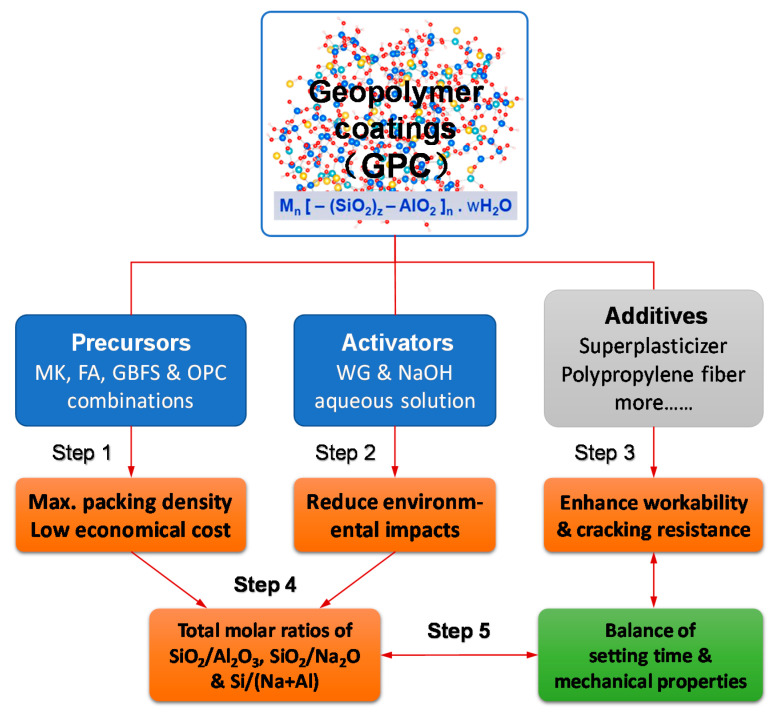
Mixture design procedure of GPC: a step by step methodology of mixture formulation involving particle packing, environmental impacts, engineering performance, as well as material chemistry.

In order to achieve proper performance of end product of GPC, the synergy of precursor and alkaline activator requires to be fully taken into account. The procedures of mixture proportioning of GPC are schematically presented in Figure 3. For the sake of achieving the balance between setting time and mechanical strength, total molar ratios of SiO_2_/Al_2_O_3_, SiO_2_/Na_2_O and H_2_O/Na_2_O as well as Si/(Na+Al) in the GPC system were calculated and regulated. According to Davidovits [39,40] and Rangan [41], the geopolymer pastes with these molar ratios in the ranges of 3.3–4.5, 0.8–2.2 and 10–25 are suitable for usage of protective coatings, respectively. The additives such as SP, PPF and retarder were adopted to further improve the particular performance of the GPC mixtures. Due to the mass proportion of the precursor being predominant, the economic cost and sustainability of GPC depend on the quadruple compositions of precursors. Less MK and more FA were suggested.

Based on the principles of mixture design, a series of GPC mixtures (85 groups) with trial and error were examined. The mixtures were listed in Table 2. As stated previously, the GPC mixtures containing multi-componential precursors were highlighted. It should be noted that, in Section 3, the experimental results will be interpreted in terms of various factors that affected the properties rather than serial numbers of the GPC mixtures.

### 2.4. Mixture Preparation

The preparation of the GPC mixtures in the laboratory consists of the following four steps. First of all, prescribed quantities of WG and NaOH were carefully blended and dissolved in the mixing water to obtain the activator solution with the required concentration. The desired concentration (or molarity) of NaOH is suggested to be within the range of 8–16 mol/L. However, 8–10 mol/L is adequate for most cases [38,42]. Too high alkalinity is prone to result in the efflorescence [43] and safety hazard of operations. The activator solution was cooled down and stabilized for at least 24 h before further mixing with the precursors. Secondly, the prescribed MK, GBFS, FA, and OPC combinations were fully mixed together to ensure the uniformity using a mechanical mixer. In the third step, the formulated alkaline solution was fully mixed with the specific additives. At this point, the liquid and solid components of the GPC mixture were prepared. Finally, the solid component was added to the liquid one and then the mixing process was performed to obtain the fresh GPC mixtures. All these steps were performed with constant temperature and relative humidity (RH) of 25 °C ± 2 °C, 60% ± 5%, respectively.

### 2.5. Experimental Procedures

Initial and final set of GPC mixtures was determined with Vicat needle method, which is in accordance with ASTM C191 [44] with necessary modifications [45]. The method requires casting the fresh mixture into a truncated ring with a height of 40 mm. The measurement started after 10–15 min of curing the GPC samples in an environmental chamber (25 °C ± 2 °C and 95% ± 5% RH) and continued with an interval of 5–15 min up to the final set.

Compressive strength of GPC was performed on 20 mm cubes at the ages of 3, 7 (or 10) and 28 days, respectively. The compacted specimens (six replicates for each test) with the steel molds were initially stored in the above-mentioned chamber (standard condition) until 24 h and then stripped, and placed again in standard condition up to the measured ages. The tests were performed on a mechanical testing machine with a capacity of 300 kN. To examine the latent influence of curing condition on compressive strength, a comparison group for each GPC mixture was continuously immersed in saline water until the test ages. The saline water was prepared with crude sea salt of a 7% concentration that is an analogue of marine environment.

**Figure 4:**
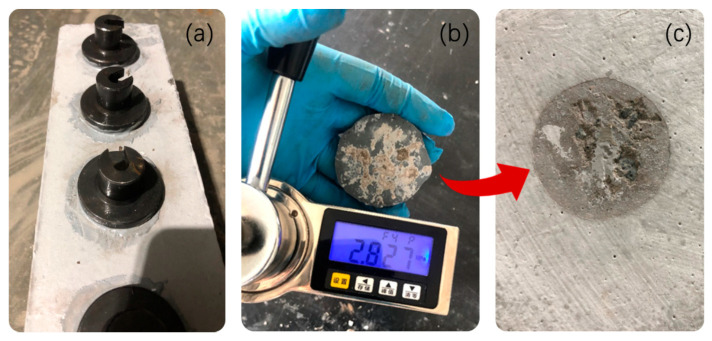
Adhesive strength test of GPC: (**a**) standard steel flanges glued on the surface of GPC layer that was previously coated on concrete substrate; (**b**) pulling out steel flanges from glued GPC surface with a portable adhesion tester, the LCD screen displays adhesive strength (MPa) of GPC; (**c**) failure interface between GPC layer and concrete substrate.

Adhesive strength (more accurately, pull-off adhesion strength) of GPC was determined using a portable adhesion tester (HCTC-10, provided by Beijing HICHANCE Technology Co., Ltd. in China). This test method covers procedures for evaluating the adhesion of a coating on concrete. The test determines the greatest perpendicular force in tension that a surface area can bear before a plug of material is detached. Failure will occur along the weakest plane within the system comprised of the loading fixture, glue, coating system, and substrate, and will be exposed by the fracture surface. The fresh GPC was painted and coated on the surface of concrete substrate (with an area of 100 mm × 400 mm) that was previously polished and handled to a saturated and surface dry state. The thickness of GPC layer was 3 mm. As shown in Figure 4, after being exposed to standard conditions up to 5 and 26 days, five standard steel flanges with Φ50 mm smooth surface were glued on the GPC layer with epoxy resin (Figure 4a). After the resin was fully cured (~48 h), the flanges were detached using a portable tester and adhesive strength was displayed on the LCD screen (Figure 4b). The failure interface in Figure 4c indicated that adhesive strength of GPC is comparably higher than that of the mortar part in concrete. In addition, the concrete substrate used was previously fabricated with the following mixture proportion (kg/m^3^): 255 kg OPC, 206 kg FA, 846 kg river sand, 928 kg crushed stone, 160 kg water, and 9.5 kg SP. The concrete substrate was cast and cured in standard condition until the age of 28 days. The characteristic value of compressive strength is 40 MPa.

As the initial attempt, chloride permeability of GPC characterized through charge passed (Coulombs) was tested with a rapid method as per ASTM C1202 standard [46]. One cross section of the protected cylinder concrete specimen (Φ100 × 50 mm) was previously polished and coated with the GPC mixture to be examined. At the age of 28 days, the charge passed of the coated concrete specimen was determined with a specialized apparatus [46] in parallel with the control one without GPC.

## 3. Results and Discussion

### 3.1. Setting Time

The GPC mixtures solely prepared with GBFS precursor often give high early-age strength but very rapid set within 30 min. Commercial retarders for OPC have been proven ineffective in geopolymer systems [47]. Lv and Cui [30] reported that an addition of polycondensed aluminum phosphate can prolong the solidification of an inorganic geopolymer-based coatings. However, the authors were unable to achieve the observation in this study. It seems as if there is a dilemma between strength development and setting time of GPC. Replacing GBFS with other aluminosilicates and formulating multi-component precursors can prolong setting time to great extent. In this section, we will present and interpret a series of factors affecting the GPC’s setting time.

#### 3.1.1. Effect of Activator Composition

**Figure 5:**
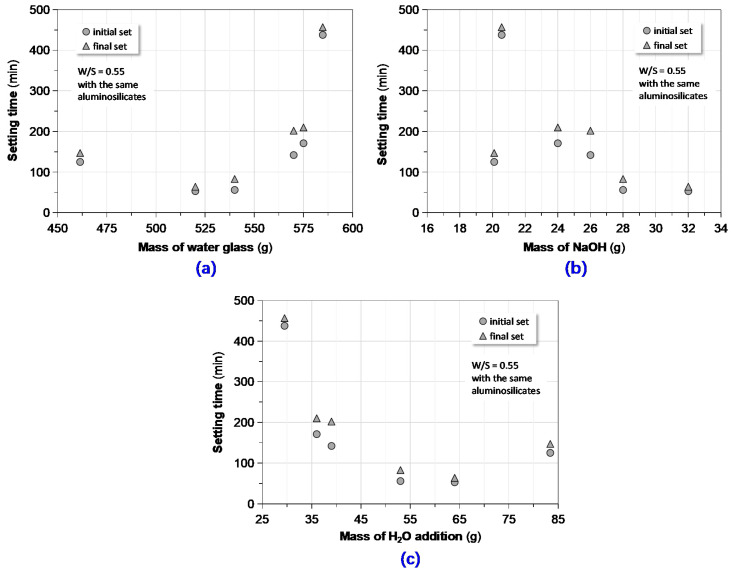
Correlating setting time to activator solutions of GPC: (**a**) setting time vs. amount of water glass; (**b**) setting time vs. amount of NaOH; (**c**) setting time vs. amount of additional mixing water.

The influence of the alkaline activator on setting time of GPC mixtures can be separately explored from its triple compositional makeup, viz., the mass of water glass, NaOH and additional water. The experimental results are clearly plotted in Figure 5 respectively. In these scatter diagrams, the test data is based on the identical precursor combinations (MK = 125 g, FA = 225 g, GBFS = 125 g and OPC = 25 g) and the constant water-solid ratio (W/S = 0.55).

In general, the relationships between the setting time and the activator composition were not quite monotonous. The intervals between the initial and final setting time of different mixtures were not long enough as those of the Portland cement paste did. However, for a first approximation, more water glass led to the extension of setting time while an increase of the quantity of NaOH or the mixing water resulted in a decrease of setting time, respectively. Keep in mind that the alkalinity and the pH value of NaOH is much higher than water glass; their contributions on the activation and geopolymerization are different; and in turn the influence of setting time is somewhat distinguished. With regard to the mixing water, its impact manner and degree cannot seem to be explained with the dilution effect that is applicable to cement-based materials, even though the polycondensation of geopolymer produces water molecules [15,17,23,29].

As far as the effect of the precursors on setting time was concerned, it was observed that the style or mode of action of each component in the quadruple precursor failed to come to any agreement. Due to the fact that GPC is a complex mixture system, we should interpret physical mechanisms of various factors on its property from a more comprehensive viewpoint. Maybe, replacing the single components as impact factors with the total quantities/moles of specific oxides or chemical elements in the entire GPC system is more reasonable [23,26].

#### 3.1.2. Effect of Key Molar Ratios

**Figure 6:**
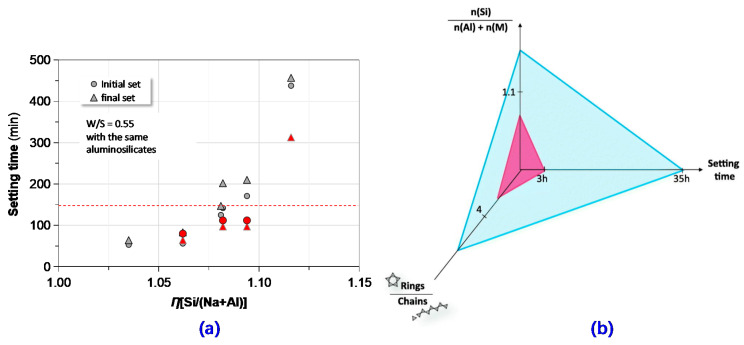
Correlating *η*[Si/(Na+Al)] to setting time of GPC: (**a**) setting time vs. *η*[Si/(Na+Al)] in this study alongside the reported data; (**b**) setting time vs. *η*[Si/(Na+Al)] reported in previous literature [48].

**Figure 7:**
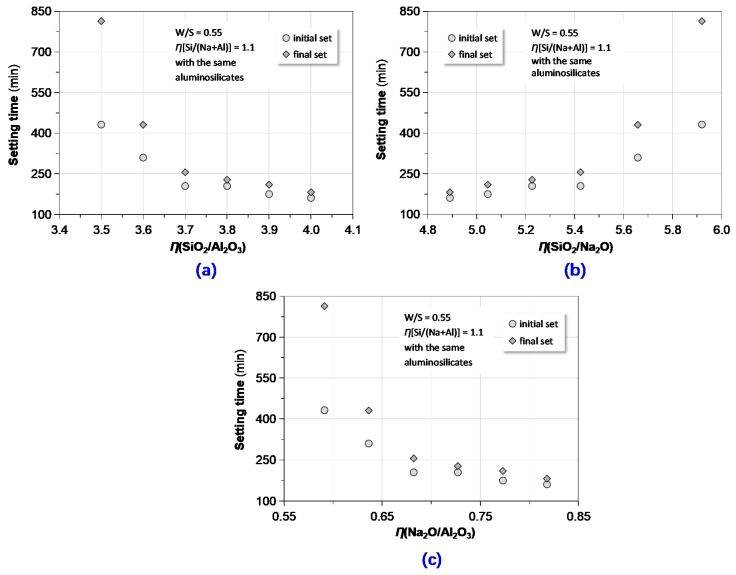
Correlating setting time to key molar ratios of GPC: (**a**) setting time vs. *η*(SiO_2_/Al_2_O_3_); (**b**) setting time vs. *η*(SiO_2_/Na_2_O) and (**c**) setting time vs. *η*(Na_2_O/Al_2_O_3_).

In this paper, the total molar ratios of several key oxides (i.e., SiO_2_, Na_2_O, Al_2_O_3_, and H_2_O) or chemical elements (i.e., Si, Na and Al) are denoted as *η*(SiO_2_/Na_2_O), *η*(SiO_2_/Al_2_O_3_), *η*(Na_2_O/Al_2_O_3_), *η*(H_2_O/Na_2_O), and *η*[Si/(Na+Al)]. In Figure 6 and Figure 7, the authors tried to correlate setting time of the GPC mixtures to the total molar ratios of these oxides or elements. *η*(SiO_2_/Na_2_O) and *η*(SiO_2_/Al_2_O_3_) separately represented the alkali and silica contents in the GPC system.

According to the interrelation among setting time, *η*[Si/(Na+Al)] and alkaline silicate’s molecule structure (ring or chain) of MK-based geopolymer developed by Arnoult et al. [48], as shown in Figure 6b, with a reference value of *η*[Si/(Na+Al)] = 1.1, were selected to explore a potential relationship between setting time and *η*[Si/(Na+Al)] of the GPC mixtures. The results are depicted in Figure 6a along with the data points collected from preceding studies [47,48]. It was obvious that the setting time was positively correlated with *η*[Si/(Na+Al)]. Once the value of *η*[Si/(Na+Al)] exceeded 1.1, an abrupt increase of setting time occurred. The default value of *η*[Si/(Na+Al)], which corresponds to favorable setting time (100–200 min), was seemingly suitable for the current coating applications.

Keep a constant value of *η*[Si/(Na+Al)] = 1.1 and the identical precursors; the relationships between setting time and the above-mentioned total molar ratios of key oxides are presented in Figure 7. Apparently, compared to the preceding correlations concerning setting time and single components, the setting time-total molar ratio relationships were definite and straightforward. It is also worth noting that the interval between initial and final setting time gradually increased with a decrease of *η*(SiO_2_/Al_2_O_3_) or *η*(Na_2_O/Al_2_O_3_) as well as an increase of *η*(SiO_2_/Na_2_O), respectively.

Ozer and Soyer-Uzun [49] characterized the effect of *η*(Si/Al) on reaction products of alkali-activated MK with X-ray diffraction (XRD) and SEM technology. They found that the geopolymer product transformed from crystalline phase (zeolite-A) to amorphous microstructures (N-A-S-H gel) with the increase of *η*(Si/Al). The observation was recently further supported by Chen et al. [50]. The extension of setting time with the reduction of *η*(SiO_2_/Al_2_O_3_) was probably ascribed to this phase transformation, but the latent mechanism is still unclear. In the present usage of geopolymer, in order to obtain proper setting behavior, the appropriate *η*(SiO_2_/Al_2_O_3_) should be selected from the range of 3.7–4.0.

#### 3.1.3. Effect of Preparation Method

**Figure 8:**
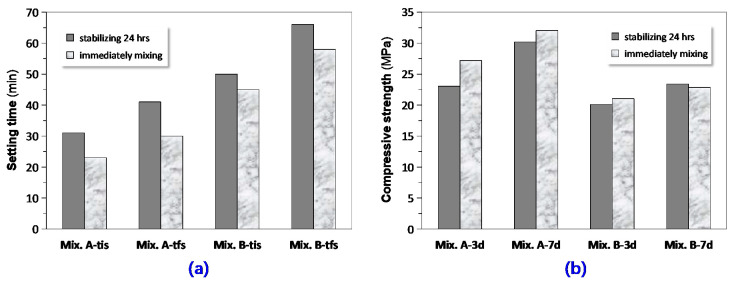
Influence of preparation procedure on basic properties of GPC: (**a**) setting time (tis = initial setting and tfs = final setting); (**b**) compressive strength at 3 and 7 days. In the two graphs, Mix. A and B stand for the GPC mixtures randomly extracted from the investigated mixtures.

It was generally suggested that the blended activator solution (water glass + alkali metal hydroxides + H_2_O) should be prepared at least 24 h in advance followed by mixed with the precursors. However, the reason why this emphasizes the procedure was not definitely stated. We took two sets of experiments randomly on two GPC mixtures to examine the effect of preparation of alkali-activator on basic properties such as setting time and compressive strength. In the control test, the activator was made as per the common procedures. Meanwhile, the mixed activator solution was immediately blended and stirred with the precursors in the parallel test. The results presented in Figure 8 indicated that the activator after stabilizing 24 h led to a more prolonged setting time than its immediately mixed counterpart did. In other word, after the storage of a period of time, the reactivity of the activator reduced to some extent. This assertion would also be explained from the results of early-age mechanical strength of the both GPC mixtures made from the activators with different initial states: the GPC mixtures made from the immediately mixed activator obtained higher compressive strength at the ages of 3 and 7 days. The effects of the preparation method on the strength and setting time were also dependent upon the precursor combinations along with the specific age. The effect on the strength was more obvious at an earlier age [35,44].

### 3.2. Compressive Strength

Mechanical strength of geopolymer presented in the literature varied widely because of the specific attributes of constitute materials, methods of preparation and exposed conditions [51]. However, there is very limited research that has been performed on comprehensive evaluation of the influencing parameters. Thermal curing is often difficult to achieve and inconvenient for in-situ applications; therefore, it is desirable to develop a GPC that can come into effect at ambient temperature. Furthermore, what we found was that, in comparison with the influence of alkaline activator, the dependence between compressive strength and precursor composition of the GPC mixtures was much stronger and more robust.

#### 3.2.1. Effect of Precursor Composition

As the binder of the geopolymer, the precursors and their make-up are of vital importance to strength of GPC. Since GBFS, FA and MK have been widely utilized to synthesize geopolymers, we proposed a blended precursor system involving these typical aluminosilicates along with an addition of OPC, supposing that their advantages are complementary. The respective proportions of these aluminosilicates in the quadruple precursor system were intended to optimize on the basis of compressive strength, more accurately the balance of strength and setting time of the GPC mixtures [21,35,44].

**Figure 9:**
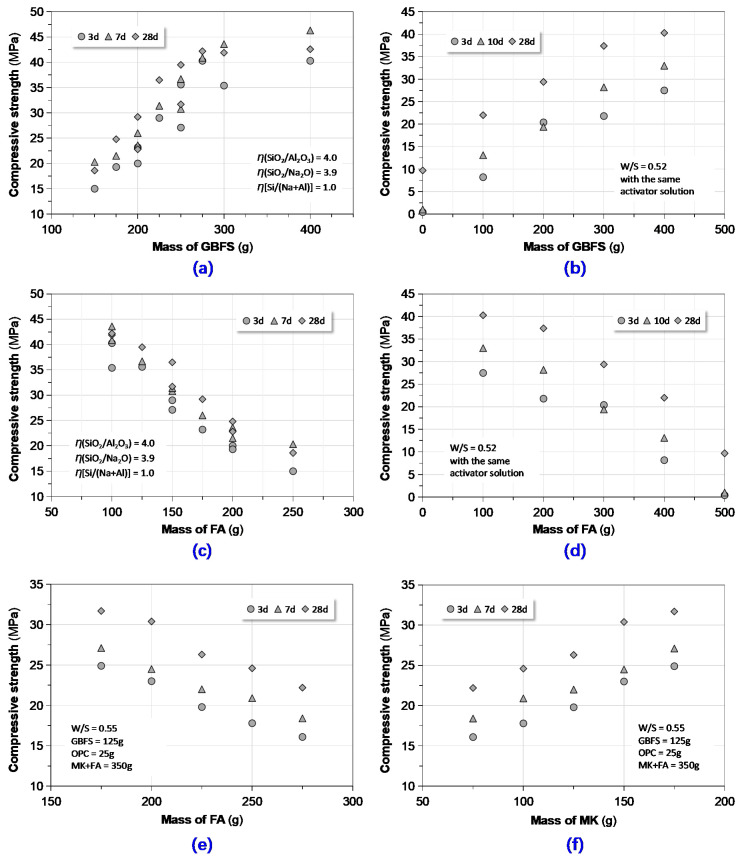
Correlating compressive strength to quadruple precursor composition of GPC: (**a**,**b**) compressive strength vs. mass of GBFS; (**c**–**e**) compressive strength vs. mass of FA; and (**f**) compressive strength vs. mass of MK.

As shown in Figure 9, compressive strength of the GPC at various ages (3, 7, 10 and 28 days) was positively and negatively affected by the mass content of GBFS and FA in the quadruple precursor, respectively, regardless of activator’s composition, W/S and curing condition. Keeping identical mass of (MK + FA) and GBFS, with an increase of mass content of MK, the GPC’s compressive strength increased. The influences of these binder components on compressive strength strongly pertain to their chemical reactivity. The mass content ranges of the precursor components were normalized on the one hand by the particle packing and were limited on the other hand by the attributes. For instance, more than 35% MK will result in an increase of mixing water while more than 50% GBFS will lead to very rapid set. Therefore, when one formulates the GPC mixture, it is critical to balance the strength and setting properties.

#### 3.2.2. Effect of Key Molar Ratios

According to our observations, the effects of key molar ratios on compressive strength were less evident than on setting time did. With regard to the influences of *η*(SiO_2_/Na_2_O) and *η*(SiO_2_/Al_2_O_3_), several investigations have been carried out. Cheng et al. reported that the MK-waste catalyst-based geopolymer samples with higher *η*(SiO_2_/Na_2_O) appeared to possess more compact structures, higher strength and lower porosity [52]. Gao et al. highlighted that the nano-SiO_2_ and MK-based geopolymer with the *η*(SiO_2_/Na_2_O) value of 1.5 exhibited higher strength and less porosity [53]. Wang et al. presented a comprehensive study on molecular structure and mechanical properties of geopolymers with different *η*(SiO_2_/Al_2_O_3_) by means of experiments and simulation. They indicated that, with the increases of *η*(SiO_2_/Al_2_O_3_), both the stability of molecular structure and mechanical properties of resultant geopolymer decreased [31]. Various zeolites were main products in geopolymers with a relatively low *η*(SiO_2_/Al_2_O_3_) = 2.0–2.5. The formation of these zeolitic structures resulted in low-strength MK-based geopolymers [54]. These findings basically coincided with the relevant results in this work, so there is no need to repeat.

**Figure 10:**
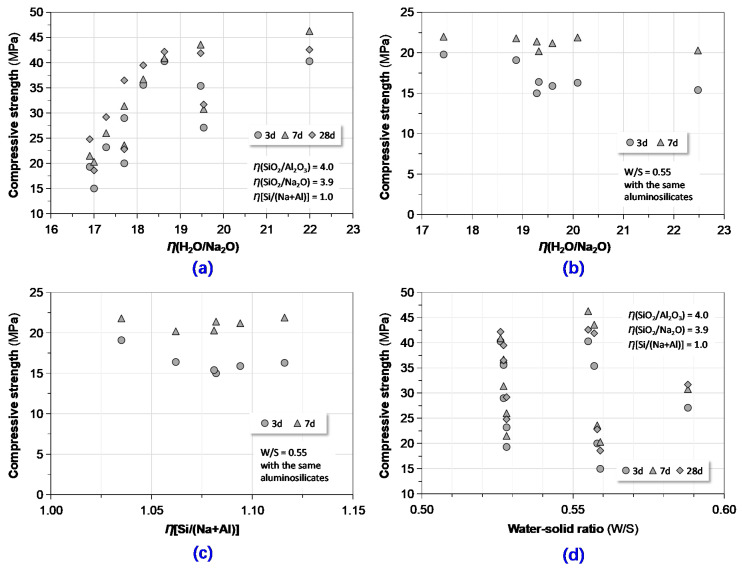
Correlating compressive strength to key molar ratios and W/S: (**a**,**b**) compressive strength vs. *η*(SiO_2_/Na_2_O); (**c**) compressive strength vs. *η*[Si/(Na+Al)]; and (**d**) compressive strength vs. W/S.

In the case of the effects of *η*(H_2_O/Na_2_O), *η*[Si/(Na+Al)] and W/S, the corresponding experimental results are plotted in Figure 10. With the identical *η*(SiO_2_/Na_2_O), *η*(SiO_2_/Al_2_O_3_) and *η*[Si/(Na+Al)], the correlation between compressive strength and W/S was elusive. The observation was basically different from the findings reported by Juengsuwattananon et al. who indicated that the geopolymer’s strength decreased with an increase of W/S [54]. They ascribed the phenomenon to dilution effect by adding more water to the system. More mixing water led to a reduction in pH value of the system and adversely affected the reaction rate. In nature, W/S or liquid-solid ratio are the practical forms of *η*(H_2_O/Na_2_O). However, the influences of *η*(H_2_O/Na_2_O) on strength varied with experimental configurations. When *η*(SiO_2_/Na_2_O), *η*(SiO_2_/Al_2_O_3_) and *η*[Si/(Na+Al)] were held constant, higher *η*(H_2_O/Na_2_O) led to greater strength. Otherwise, when the same precursors were used, the strength slightly decreased with the increase of *η*(H_2_O/Na_2_O). The effect of *η*[Si/(Na+Al)] was also inapparent.

#### 3.2.3. Effect of Exposed Conditions

**Figure 11:**
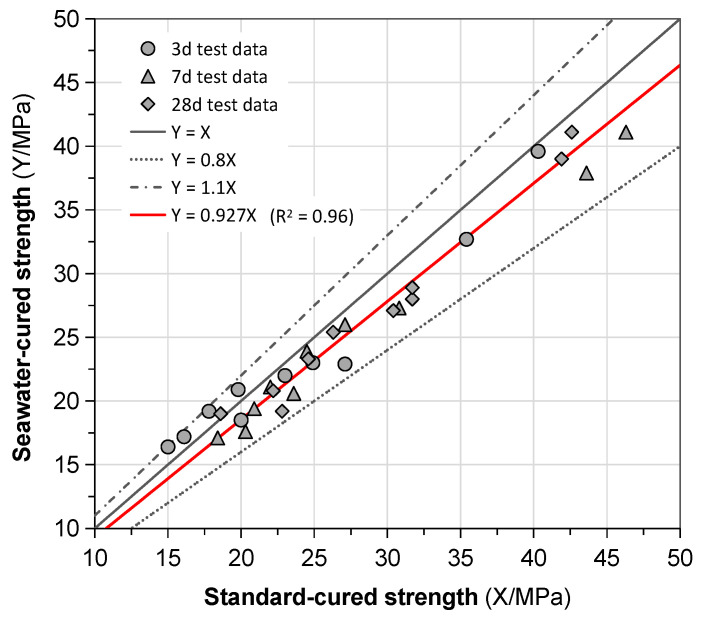
Correlating compressive strength of GPC immersed in artificial seawater to its counterpart stored in standard condition.

In Figure 11, we compared the standard cured and seawater-immersed GPC’s compressive strength. It was easy to find that, although continuous seawater immersion impaired the strength, the loss of 7% was more or less negligible from a viewpoint of practical applications. That is to say, water resistance and anti-corrosion of the GPC mixtures are desirable, even though the exposed condition is harsh. This observation coincided with the results presented by Zhang et al. [27]. The strength reduction of the seawater-immersed specimens could be attributed to dilution of excessive water rather than the corrosion of seawater. Water reservoir also inhibited the polycondensation to some extent [36,51]. Due to excellent impermeability of the GPC, the dilution effect was limited to the surface layer.

### 3.3. Several Critical Observations

With the exception of all above-discussed factors that affected basic properties of GPC, there are some critical experimental results that are worthwhile to discuss or interpret here.

#### 3.3.1. A Threshold of Total Molar Ratio

In this subsection, with the same W/S, WG and precursor combination, the relationship between setting time/early-age strength and mass of NaOH/*η*(SiO_2_/Na_2_O) was discussed. In this experimental configuration, the only independent variable was the mass of NaOH.

**Figure 12:**
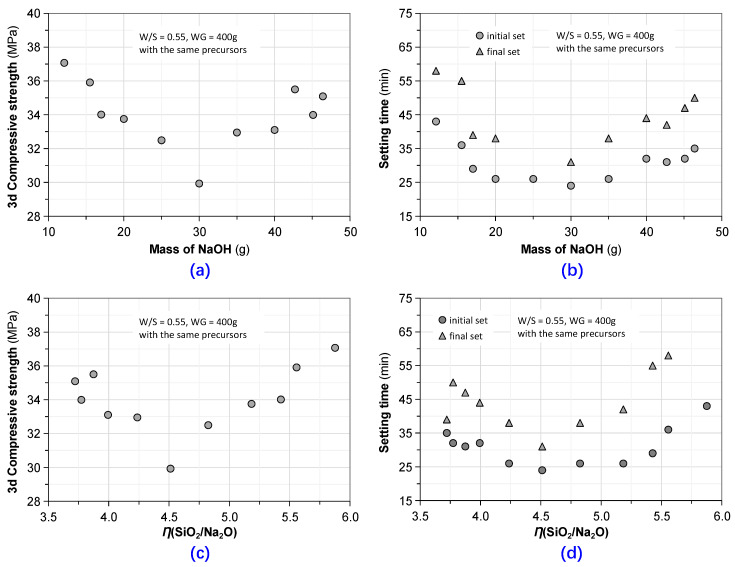
A critical observation on setting time and compressive strength: (**a**,**b**) compressive strength at the age of 3 days and setting time vs. the mass of NaOH; (**c**,**d**) compressive strength at the age of 3 days and setting time vs. *η*(SiO_2_/Na_2_O), respectively.

From a phenomenological perspective, it seems as if there was a threshold of the mass of NaOH (30 g) that divided its influence on the strength as well as setting time into two distinct segments (see Figure 12a,b). In the first part, the setting time and strength decreased with an increase of the mass of NaOH, and in the second part, the reverse happened. Because *η*(SiO_2_/Na_2_O) consistently decreased with the increasing addition of NaOH, the correlations between setting time or early-age strength and *η*(SiO_2_/Na_2_O), as shown in Figure 12c,d, highly resembled one another. The threshold of *η*(SiO_2_/Na_2_O) equals to 4.56. Hamidi indicated that the geopolymer paste activated with 12 M NaOH solution obtained the greatest mechanical strength in an experimental study exploring the effect of molarity of NaOH (4–18 M) [55]. In addition, the molarity or content of NaOH in the alkali-activator affected the fluidity of the fresh GPC mixtures and solid particle size [56]. Higher concentration of NaOH solution often leads to better flowability and less particle agglomeration. Similar test results were also observed in this research. The effects of the concentration of alkali metal hydroxide on properties of geopolymer are dependent on the change of zeta potential in the alkaline activator [57].

#### 3.3.2. Effect of OPC Addition

In the binder of geopolymer, OPC, which can cause environmental load such as CO_2_ emission, is often not recommended. However, as an important calcium source, the hydration of OPC has two roles: accelerate geopolymerization and produce calcium silicate hydrate (C-S-H) gel [58]. The coexistence of sodium aluminosilicate hydrate (N-A-S-H) and C-S-H will enhance the engineering properties of geopolymer-based materials [59]. In the quadruple precursor, the dosage of OPC should be determined in accordance with the balance between compressive strength and setting behavior of the prepared GPC.

**Figure 13:**
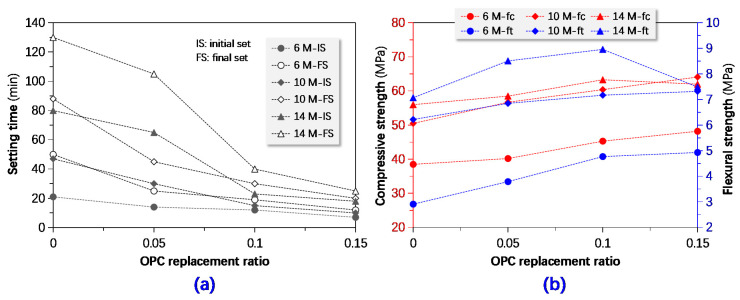
Influences of ordinary Portland cement (OPC) replacement ratio (by mass) on (**a**) setting time and (**b**) compressive and flexural strength.

As clearly depicted in Figure 13, setting time and strength properties separately shortened and raised with the increase of OPC replacement ratio in general. Besides, the effects were implicated with the molarity of NaOH in the activator solution; the higher the molarity, the stronger the effects. When the mass ratio of OPC was greater than 10% by total mass of precursor, the GPC’s strength tended to decrease, especially the ones activated with higher concentration NaOH solution. Other calcium-rich compounds such as calcium hydroxide or calcium oxide can also be utilized to regulate setting time and mechanical properties; their replacement ratios should be tailored in terms of particular usages [16,22,34].

#### 3.3.3. Effect of GBFS Type

**Figure 14:**
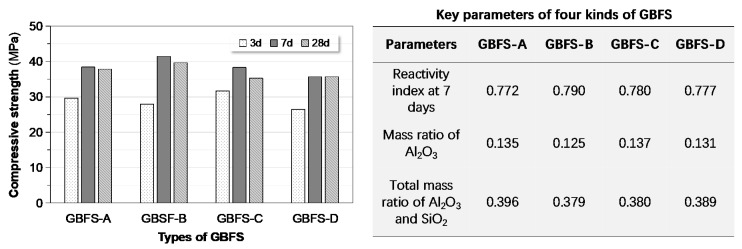
Correlating compressive strength to types of GBFS: several key parameters in terms of reactivity of GBFS are listed alongside.

The properties of geopolymer are affected not only by mixture proportions of precursor and activator, but also by type and source of constituent materials. Figure 14 shows the compressive strength of GPC mixtures prepared from four different GBFS with the same recipes. GBFS-B and GBFS-D separately produced the highest and the lowest strength. It appears that this observation was not easy to interpret from key parameters of GBFS such as reactivity index, Al_2_O_3_ content or total content of Al_2_O_3_ and SiO_2_. The geopolymerization process is complicated and related with the phase morphology of the precursors apart from their combinations and oxide compositions. By the way, the influence of type of GBFS on setting behavior was also ignorable.

At this point, the optimized 5 GPC mixtures presented in Table 3 with both practicable setting time (not earlier than 120 min) and desirable compressive strength (not less than 35 MPa) were selected from the investigated 85 mixtures listed in Table 2. In the course of seeking the optimized mixtures, a series of factors affecting the basic properties of GPC were explored and discussed. In particular, an eye was kept on the balance between strength and setting behavior at ambient temperature as well as the usage of quadruple precursors.

### 3.4. Adhesive Strength

The adhesive strength of the optimized GPC mixtures was measured through a portable tester, as listed in Table 3. All the test results were higher than 1.5 MPa, regardless of the measured age. Previous investigations indicated that the adhesive strength of coatings for most civil applications should not be less than 1.4 to 1.75 MPa, which varied from one testing method to another. According to the provisions in Chinese technical standards, the adhesive property of theses mixtures fully meets the requirement of surface protection. It is easy to understand that higher compressive strength often responds to higher adhesive strength.

### 3.5. Rapid Chloride Permeability

**Figure 15:**
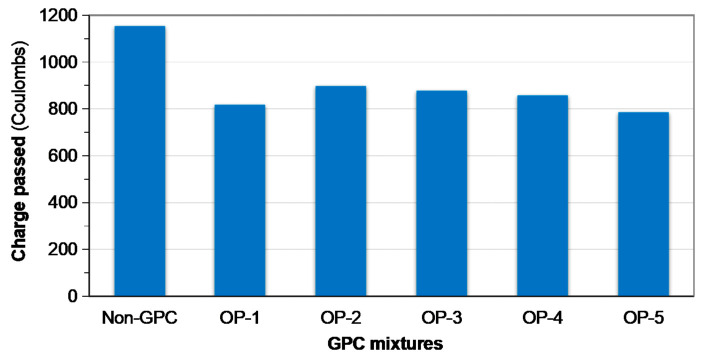
Rapid chloride permeability of coated and uncoated concrete substrate characterized through accelerated charge passed: “Non-GPC” stands for uncoated concrete substrate, and “OP-x” stands for the optimized GPC mixtures listed in Table 3.

In order to characterize the compactness of GPC protective layer, as an easily determined indicator, charge passed based on rapid chloride permeability test was explored herein. The preferred mixtures were separately applied to the concrete substrate, and their respective permeability was compared to that of the naked substrate in Figure 15. Apparently, the coated substrate with the GPC obtained improved resistance to deleterious water-soluble ions. The average charge passed of the coated concrete specimens was approximately 30% lower than that of the uncoated ones. The transport mechanism of chemical species in the geopolymer will be further studied.

### 3.6. An Outlook on Sustainability

Apart from the engineering properties, the cost, availability and environmental compatibility of GPC are also critical to its potential application. The complexity of aluminosilicate sources, especially aluminosilicate-containing industrial by-products, is a major concern for GPC production. The multi-componential composite precursor is superior to the single precursor, from the perspective of comprehensive utilization of various resources. Due to strong corrosivity and consequent operation safety of alkali metal hydroxides, certain alkaline by-products from chemical industry are more suitable to prepare GPC. Moreover, the end product of GPC possesses preferred life-cycle performance as opposed to organic coating materials. Several authors calculated carbon footprint (or emission factor) and economic cost as well as embodied energy consumption of geopolymer-based product in terms of laboratory or industrial scale [60,61,62,63]. Although these analyses referred to different original databases, the results generally indicated that geopolymer is a sustainable alternative material. The preparation of GPC relies on the alkali-activation and the subsequent polycondensation without extra inputs [6]. This process kills two birds with one stone by meeting usage requirements as well as by achieving a cleaner future [64].

## 4. Conclusions

Apart from as a partial alternative to Portland cement, this paper reports an experimental study on designing, preparing and characterizing a cluster of sustainable GPC that can be potentially utilized to surface the protection of civil infrastructures. Based on the results presented, the following conclusions can be drawn:

- Optimized GPC mixtures with both reasonable setting time and desirable compressive strength were selected from the investigated 85 mixtures. Balancing strength and setting behavior at ambient temperature was viable with the aid of quadruple precursors and mixture design.
- Adhesive strength of preferred GPC mixtures was successfully measured with a portable tester. The test results ranged from 1.5 to 3.4 MPa and fully meet the requirements of surface protection.
- Characterizing GPC’s permeability based on a rapid test is feasible and easy to handle. The charge passed of coated concrete specimens with the optimized GPC was 30% lower than that of the uncoated ones.
- Setting time of GPC was positively correlated with *η*[Si/(Na+Al)]. When the value was greater than 1.1, an abrupt increase of setting time occurred. The extension of setting time with the reduction of *η*(SiO_2_/Al_2_O_3_) was probably ascribed to phase transformation of GPC.
- Compressive strength of GPC was positively affected by the mass contents of GBFS, MK and OPC, and was negatively affected by mass content of FA in the quadruple precursor, respectively. Sustained saline water immersion impaired the strength of GPC to a very limited extent.
- There existed a threshold of mass of NaOH that divided its influence on GPC’s strength and setting time into two distinct segments; in the first part, setting time and strength decreased with an increase of NaOH mass, and in the second part, the reverse happened.
- Replacing mass contents of the single components with the total molar ratios of specific oxides or chemical elements as design parameters in the overall GPC system is more reasonable.
- GPC is an innovative material for surface protection and a viable alternative to conventional synthetic polymer coatings for use in civil engineering applications.

## Figures and Tables

**Table 1:** Chemical compositions of precursors for geopolymer coatings.

Precursors	Oxide Compositions (Mass Fraction, %)									
	SiO_2_	Al_2_O_3_	Na_2_O	CaO	Fe_2_O_3_	MgO	TiO_2_	K2O	H_2_O	LOI
MK	45.12	42.40	0.15	9.11	0.76	0.09	1.37	0.19	0.12	0.35
FA	37.00	31.88	0.66	9.11	8.35	1.16	1.81	1.29	0.13	0.46
GBFS-A ^1^	26.08	13.51	0.26	45.66	0.45	8.53	0.67	0.40	0.21	0.22
GBFS-B ^1^	25.36	12.49	0.29	47.38	0.45	6.31	0.89	0.35	0.31	0.43
GBFS-C ^1^	24.34	13.67	0.29	44.97	0.41	8.21	0.80	0.41	0.16	0.13
GBFS-D ^1^	25.79	13.11	0.26	46.28	0.77	7.00	0.95	0.42	0.11	0.25
OPC	17.27	6.63	0.25	58.25	6.38	2.69	0.51	0.74	0.16	0.42

^1^ GBFS-A, B, C, and D with roughly the same chemical compositions come from different iron works.

**Table 2:** Mixture proportions of geopolymer coatings.

Mixture No.	Precursors (g)				Activators (g)			Additives (g)		W/S	Total Molar Ratios				
	MK	FA	GBFS	OPC	WG	NaOH	W	SP	PPF		SiO_2_/Al_2_O_3_	SiO_2_/Na_2_O	Na_2_O/Al_2_O_3_	Si/(Na+Al)	H_2_O/Na_2_O
01	250	0	0	250	355.1	53.6	84.5	7.50	0.25	0.450	3.513	3.417	1.028	0.866	13.86
02	250	250	0	0	355.1	53.6	84.5	7.50	0.25	0.450	3.195	4.136	0.772	0.901	13.55
03	250	250	0	0	355.1	53.6	132.5	7.50	0.25	0.520	3.195	4.136	0.772	0.901	15.66
04	250	125	0	125	355.1	53.6	132.5	7.50	0.25	0.520	3.330	3.781	0.881	0.885	15.84
05	125	250	0	125	355.1	53.6	132.5	7.50	0.25	0.520	3.881	3.672	1.057	0.943	15.64
06	250	0	250	0	355.1	53.6	132.5	7.50	0.25	0.520	3.349	3.665	0.914	0.875	15.81
07	250	0	250	0	355.1	53.6	132.5	7.50	0.25	0.520	3.349	3.665	0.914	0.875	15.81
08	250	0	250	0	355.1	53.6	132.5	7.50	0.25	0.520	3.388	3.685	0.919	0.883	16.00
09	250	0	250	0	355.1	53.6	132.5	7.50	0.25	0.520	3.286	3.650	0.900	0.865	16.00
10	250	0	250	0	355.1	53.6	132.5	7.50	0.25	0.520	3.363	3.703	0.908	0.881	16.01
11	0	500	0	0	355.1	53.6	132.5	7.50	0.25	0.520	3.011	3.699	0.814	0.830	15.56
12	0	400	100	0	355.1	53.6	132.5	7.50	0.25	0.520	3.271	3.574	0.915	0.854	15.63
13	0	300	200	0	355.1	53.6	132.5	7.50	0.25	0.520	3.610	3.447	1.047	0.882	15.71
14	0	200	300	0	355.1	53.6	132.5	7.50	0.25	0.520	4.068	3.320	1.225	0.914	15.79
15	0	100	400	0	355.1	53.6	132.5	7.50	0.25	0.520	4.722	3.191	1.480	0.952	15.88
16	50	200	200	50	355.1	53.6	132.5	7.50	0.25	0.520	3.755	3.390	1.108	0.891	15.81
17	0	200	200	100	355.1	53.6	132.5	7.50	0.25	0.520	4.202	3.203	1.312	0.909	15.80
18	0	300	100	100	355.1	53.6	132.5	7.50	0.25	0.520	3.695	3.331	1.109	0.876	15.72
19	0	100	300	100	355.1	53.6	132.5	7.50	0.25	0.520	4.944	3.073	1.609	0.948	15.88
20	0	400	0	100	355.1	53.6	132.5	7.50	0.25	0.520	3.327	3.458	0.962	0.848	15.64
21	0	0	400	100	355.1	53.6	132.5	7.50	0.25	0.520	6.135	2.942	2.085	0.994	15.96
22	125	125	125	125	355.1	53.6	132.5	7.50	0.25	0.520	3.661	3.400	1.077	0.881	15.89
23	50	200	150	100	355.1	53.6	132.5	7.50	0.25	0.520	3.804	3.332	1.142	0.888	15.81
24	100	200	150	50	355.1	53.6	132.5	7.50	0.25	0.520	3.462	3.519	0.984	0.873	15.82
25	50	150	150	150	355.1	53.6	132.5	7.50	0.25	0.520	4.118	3.209	1.283	0.902	15.85
26	75	250	150	25	525.1	45.5	76.1	7.50	0.25	0.559	4.000	3.894	1.027	0.987	17.00
27	75	200	200	25	466.2	44.9	100.9	7.50	0.25	0.558	4.000	3.897	1.026	0.987	17.70
28	75	150	250	25	407.4	44.3	146.9	7.50	0.25	0.588	4.000	3.899	1.026	0.987	19.54
29	75	100	300	25	348.5	43.8	150.4	7.50	0.25	0.557	4.000	3.902	1.025	0.988	19.47
30	75	0	400	25	230.8	42.6	200.0	7.50	0.25	0.555	4.000	3.911	1.023	0.989	21.99
31	75	200	175	50	459.5	44.3	81.8	7.50	0.25	0.528	4.000	3.895	1.027	0.987	16.90
32	75	175	200	50	430.1	44.0	94.7	7.50	0.25	0.528	4.000	3.897	1.026	0.987	17.28
33	75	150	225	50	400.6	43.8	107.4	7.50	0.25	0.527	4.000	3.898	1.026	0.987	17.70
34	75	125	250	50	371.2	43.5	120.2	7.50	0.25	0.527	4.000	3.899	1.026	0.987	18.14
35	75	100	275	50	341.8	43.2	132.9	7.50	0.25	0.526	4.000	3.901	1.025	0.987	18.63
36	75	100	275	50	341.8	24.3	132.9	7.50	0.25	0.542	4.000	5.034	0.795	1.114	24.04
37	75	275	125	25	466.2	44.9	94.9	7.50	0.25	0.550	3.701	3.991	0.927	0.960	17.37
38	100	250	125	25	466.2	44.9	94.9	7.50	0.25	0.550	3.657	4.024	0.909	0.958	17.40
39	125	225	125	25	466.2	44.9	94.9	7.50	0.25	0.550	3.614	4.058	0.891	0.956	17.43
40	125	225	125	25	584.8	20.6	29.5	7.50	0.25	0.550	4.000	5.046	0.793	1.116	20.09
41	125	225	125	25	520.0	32.0	64.0	7.50	0.25	0.550	3.789	4.567	0.830	1.035	18.87
42	125	225	125	25	540.0	28.0	53.0	7.50	0.25	0.550	3.854	4.734	0.814	1.062	19.32
43	125	225	125	25	570.0	26.0	39.0	7.50	0.25	0.550	3.952	4.782	0.826	1.082	19.28
44	125	225	125	25	575.0	24.0	36.0	7.50	0.25	0.550	3.968	4.877	0.814	1.094	19.59
45	125	225	125	25	461.4	20.1	83.4	7.50	0.25	0.550	3.598	5.411	0.665	1.081	22.48
46	150	200	125	25	466.2	44.9	94.9	7.50	0.25	0.550	3.572	4.091	0.873	0.954	17.46
47	175	175	125	25	466.2	44.9	94.9	7.50	0.25	0.550	3.532	4.125	0.856	0.951	17.49
48	125	240	125	10	606.5	20.4	19.9	7.50	0.25	0.550	4.000	5.045	0.793	1.116	19.78
49	75	250	150	25	0.0	100.0	300.0	7.50	0.25	0.500	2.163	2.201	0.983	0.546	12.97
50	75	250	150	25	0.0	100.0	330.0	7.50	0.25	0.550	2.163	2.201	0.983	0.546	14.26
51	125	225	125	25	584.8	23.4	31	7.50	0.25	0.550	4.000	4.890	0.818	1.100	19.54
52	125	225	125	25	554.1	21.8	43.6	7.50	0.25	0.550	3.900	5.045	0.773	1.100	20.32
53	125	225	125	25	523.4	20.1	56.2	7.50	0.25	0.550	3.800	5.226	0.727	1.100	21.23
54	125	225	125	25	492.7	18.5	68.6	7.50	0.25	0.550	3.700	5.424	0.682	1.100	22.22
55	125	225	125	25	462	16.8	81.3	7.50	0.25	0.550	3.600	5.658	0.636	1.100	23.39
56	125	225	125	25	431.3	15.2	93.9	7.50	0.25	0.550	3.500	5.920	0.591	1.100	24.70
57	50	200	200	50	414.9	24.6	106.3	7.50	0.25	0.550	4.000	4.890	0.818	1.100	22.30
58	75	100	275	50	0	0	275	7.50	0.25	0.550	2.468	97.381	0.025	1.204	22.33
59	75	100	275	50	400	40	121	7.50	0.25	0.550	4.261	3.994	1.067	1.031	19.19
60	75	100	275	50	400	35	118.5	7.50	0.25	0.550	4.261	4.237	1.006	1.062	20.23
61	75	100	275	50	400	30	116	7.50	0.25	0.550	4.261	4.512	0.944	1.096	21.40
62	75	100	275	50	400	25	113	7.50	0.25	0.550	4.261	4.825	0.883	1.131	22.69
63	75	100	275	50	400	20	110	7.50	0.25	0.550	4.261	5.184	0.822	1.169	24.19
64	75	100	275	50	450	25	91	7.50	0.25	0.550	4.485	4.711	0.952	1.149	21.62
65	75	100	275	50	450	25	91	7.50	0.25	0.550	4.485	4.711	0.952	1.149	21.62
66	75	100	275	50	500	25	69	7.50	0.25	0.550	4.709	4.612	1.021	1.165	20.68
67	75	100	275	50	550	25	47	7.50	0.25	0.550	4.933	4.526	1.090	1.180	19.87
68	75	100	275	50	600	25	25	7.50	0.25	0.550	5.157	4.450	1.159	1.194	19.15
69	75	100	275	50	600	25	25	7.50	0.25	0.550	5.157	4.450	1.159	1.194	19.15
70	75	100	275	50	400	42.7	122.8	7.50	0.25	0.550	4.261	3.874	1.100	1.015	18.71
71	75	100	275	50	400	45.1	124.1	7.50	0.25	0.550	4.261	3.773	1.129	1.001	18.28
72	75	100	275	50	400	46.4	124.8	7.50	0.25	0.550	4.261	3.721	1.145	0.993	18.06
73	75	100	275	50	400	17	108	7.50	0.25	0.550	4.261	5.427	0.785	1.193	25.18
74	75	100	275	50	400	15.5	107.8	7.50	0.25	0.550	4.261	5.557	0.767	1.206	25.77
75	75	100	275	50	400	12.1	106	7.50	0.25	0.550	4.261	5.876	0.725	1.235	27.11
76	75	100	275	50	419.9	24.4	104	7.50	0.25	0.550	4.350	4.816	0.903	1.143	22.41
77	75	100	275	50	404.3	24.2	110.8	7.50	0.25	0.550	4.280	4.868	0.879	1.139	22.83
78	75	100	275	50	400	0	99	7.50	0.25	0.550	4.261	7.384	0.577	1.351	33.41
79	75	100	275	50	450	0	77.3	7.50	0.25	0.550	4.485	6.943	0.646	1.362	30.70
80	75	100	275	50	500	0	55.4	7.50	0.25	0.550	4.709	6.586	0.715	1.373	28.50
81	75	100	275	50	550	0	33.4	7.50	0.25	0.550	4.933	6.293	0.784	1.383	26.69
82	75	100	275	50	600	0	11.5	7.50	0.25	0.550	5.157	6.047	0.853	1.392	25.17
83	75	100	275	50	400	100	154.5	7.50	0.25	0.550	4.261	2.365	1.802	0.760	12.38
84	75	100	275	50	400	5	102	7.50	0.25	0.550	4.261	6.676	0.638	1.300	30.46
85	75	100	275	50	500	85	102	7.50	0.25	0.550	4.709	2.682	1.756	0.854	13.05

**Table 3:** Adhesive strength of the optimized geopolymer coatings mixtures.

Mixture No.	Precursors (g)				Activators (g)			Additives (g)		Adhesive Strength (MPa)		Compressive Strength (MPa)	
	MK	FA	GBFS-A	OPC	WG	NaOH	W	SP	PPF	7d	28d	7d	28d
OP-1	75	100	275	50	400.0	85.0	145.8	7.50	0.25	1.763	2.096	43.7	44.0
OP-2					500.0	100.0	110.5	7.50	0.25	1.554	2.613	35.5	35.2
OP-3					419.9	24.4	104.0	7.50	0.25	1.646	2.812	35.7	52.5
OP-4					404.3	24.2	110.8	7.50	0.25	2.556	3.398	41.5	52.8
OP-5					400.0	100.0	154.5	7.50	0.25	1.684	1.870	38.5	49.5
